# Left Cardiac Remodelling Assessed by Echocardiography Is Associated with Rho-Kinase Activation in Long-Distance Runners

**DOI:** 10.3390/jcdd8100118

**Published:** 2021-09-24

**Authors:** Felipe Contreras-Briceño, Julián Vega, Jorge Mandiola, María Paz Ocaranza, Sebastián Herrera, Manuel Salinas, Rodrigo Fernández, Jorge E. Jalil, Sergio Lavandero, Mario Chiong, Paz Godoy, Pablo F. Castro, Marta Sitges, Luigi Gabrielli

**Affiliations:** 1 Advanced Center for Chronic Diseases (ACCDiS), Division of Cardiovascular Diseases, Faculty of Medicine, Pontificia Universidad Católica de Chile, Av. Sergio Livingstone #1007, Santiago 8380492, Chile; fcontrerasb@uc.cl (F.C.-B.); julianvega@gmail.com (J.V.); jmandiola.o@hotmail.com (J.M.); mocaran@uc.cl (M.P.O.); sherrerat@gmail.com (S.H.); kant_76@hotmail.com (M.S.); rodri_fernandezz@hotmail.com (R.F.); jjalil@med.puc.cl (J.E.J.); pazitag.16@gmail.com (P.G.); pcastro@med.puc.cl (P.F.C.); 2 Laboratory of Exercise Physiology, Department of Health Science, Faculty of Medicine, Pontificia Universidad Católica de Chile, Av. Vicuña Mackenna #4860, Santiago 7820436, Chile; 3 Physiology Section, Department of Cell Biology, Physiology and Immunology, Faculty of Biology, Universitat de Barcelona, Av. Diagonal #643, 08028 Barcelona, Spain; 4 Center of New Drugs for Hypertension (CENDHY), Universidad de Chile & Pontificia Universidad Católica de Chile, Av. Santos Dumont #964, Santiago 8380494, Chile; 5 Advanced Center for Chronic Diseases (ACCDiS), Faculty of Chemical & Pharmaceutical Sciences & Faculty of Medicine, Universidad de Chile, Av. Sergio Livingstone #1007, Santiago 8380492, Chile; slavander@uchile.cl (S.L.); mchiong@uchile.cl (M.C.); 6 Department of Internal Medicine, Cardiology Division, University of Texas Southwestern Medical Center, Av. Harry Hines Blvd #5323, Dallas, TX 75390-8573, USA; 7 Thorax Institute, IDIBAPS, Hospital Clinic, Av. Carrer del Rosselló #149, 08036 Barcelona, Spain; msitges@clinic.cat

**Keywords:** athlete’s heart, cardiac biomarkers, echocardiography, exercise, functional cardiac capacity

## Abstract

This single-blind and cross-sectional study evaluated the role of Rho-kinase (ROCK) as a biomarker of the cardiovascular remodelling process assessed by echocardiography in competitive long-distance runners (LDRs) during the training period before a marathon race. Thirty-six healthy male LDRs (37.0 ± 5.3 years; 174.0 ± 7.0 height; BMI: 23.8 ± 2.8; $\dot{V}$ O_2_-peak: 56.5 ± 7.3 mL·kg^−1^·min^−1^) were separated into two groups according to previous training level: high-training (HT, *n* = 16) ≥ 100 km·week^−1^ and low-training (LT, *n* = 20) ≥ 70 and < 100 km·week^−1^. Also, twenty-one healthy nonactive subjects were included as a control group (CTR). A transthoracic echocardiography was performed and ROCK activity levels in circulating leukocytes were measured at rest (48 h without exercising) the week before the race. The HT group showed a higher left ventricular mass index (LVMi) and left atrial volume index (LAVi) than other groups (*p* < 0.05, for both); also, higher levels of ROCK activity were found in LDRs (HT = 6.17 ± 1.41 vs. CTR = 1.64 ± 0.66 (*p* < 0.01); vs. LT = 2.74 ± 0.84; (*p* < 0.05)). In LDRs a direct correlation between ROCK activity levels and LVMi (r = 0.83; *p* < 0.001), and LAVi (r = 0.70; *p* < 0.001) were found. In conclusion, in male competitive long-distance runners, the load of exercise implicated in marathon training is associated with ROCK activity levels and the left cardiac remodelling process assessed by echocardiography.

## 1. Introduction

Physical exercise plays a fundamental role in cardiovascular disease prevention and significantly reduces global mortality [1]. This benefit is associated with different mechanisms linked to structural changes or “adaptation” of the heart [2]. The combination of these adaptations, known as cardiovascular remodelling, involves changes at molecular and cellular levels which translate into structural, electric and functional cardiovascular modifications [3].

The cardiac remodelling process can occur early during the training process [4]. Highly trained runners experience these changes with greater prevalence and intensity, which in most cases are benign and reversible [2]—a condition called “athlete’s heart” [5] that includes increased bi-ventricular diameter, left ventricle (LV) parietal thickness, LV mass and bi-atrial volume with systolic and normal diastolic function [6]. The majority of these changes are a physiological adaptation to exercise; however, some patterns may overlap with channelopathies or cardiomyopathies [7]; LV hypertrophy criteria are present in as many as 70% of highly trained athletes [8], and only 12% showed criteria for right ventricular hypertrophy [9].

Advances in image techniques have allowed a better characterization of athlete’s heart. It is known that these changes are related with better sports performance [10]; however, some predisposed athletes subjected to high training loads may show a potentially adverse cardiac remodelling (“Phidippides” cardiomyopathy) [6,11]. This adverse cardiac remodelling process is not frequent and is characterized by LV hypertrophy associated with myocardial fibrosis [8], increased coronary atheromatosis, greater right ventricular remodelling and extreme right and left atrial (LA) dilatation [12,13]; there is uncertainty over whether there is a maximum limit of training considered safe, and if it is possible to identify an individual limit for each athlete [13].

The physiological and eventually pathophysiological mechanisms linked to adverse cardiac remodelling in high-performance athletes are not completely clear. In this regard, the dynamics of different biomarkers related to tissue damage, inflammation, oxidative stress and cardiac remodelling have been studied in athletes of different disciplines with no clear significance [6]. A novel pathway related to cell survival and cardiac adaptation to stress is rho-kinase (ROCK) activation [14]. This kinase exerts its role by acting on the cytoskeleton, regulating cell motility (migration), adhesion, and proliferation, assuming a leading role in mediating cardiac remodelling [15,16] in different clinical scenarios [17,18]. The association of ROCK with exercise has been poorly explored. Recently, investigations have reported an increase of ROCK activity induced by aerobic exercise in the skeletal muscle of rats, linked to glucose uptake through insulin receptor substrate 1 (IRS1) phosphorylation [18,19]. In patients with dyslipidemia, moderate aerobic physical exercise (30 min × 5 days a week) has been related to attenuation of ROCK concentration [20]. The role of insulin pathways during the hypertrophic process and ROCK activation induced by exercise has been recently reported in an interesting review by Anaruma et al. [21]. However, the dynamics of ROCK activation and their association with cardiac remodelling in endurance athletes with highly demanding training protocols, as for a marathon race competition, has not been previously studied. Thus, the primary objective of this study was to evaluate the activity of ROCK and its association with cardiac remodelling assessed by echocardiography in competitive long-distance runners (LDRs) with different training loads before a highly demanding competition (marathon).

## 2. Materials and Methods

The sample size calculation was done by G*Power^®^ 3.1 software (Heinrich–Heine University, Dusseldorf, Germany) using previous data concerning the association found between echocardiography measurements (negative deformation of the post P wave strain curve, LASa) and $\dot{V}$O_2_-max in runners with similar characteristics to the participants in this study (rho = 0.546; *p* = 0.028) [10]—considering a significance level of 5% and a power of 80%, in a two-tail test, plus 10% of data loss. Thus, thirty-six Caucasian males recreational LDRs were recruited previously to a marathon race (Santiago, 42.2 km). The participants were included 16 weeks before the competition, in the training period called “optimal phase”, where the volume of training is increased by running a longer distance each week. Hydric support during preparations follows the recommendation of our sport section group: as a rule, during high intensity or hard physical activity it is recommended to have intakes of 0.6–1 L·h^−1^ with frequent intakes (150–250 mL) every 15–20 min, and always with an isotonic content; also, with frequent body weight checks. The inclusion criteria were: (i) age between 18 and 50 years to minimize possible cardiovascular events linked to the competition, (ii) participation in three or more completed marathons in the last five years, and (iii) recreational status, to obtain a more diverse sample. The exclusion criteria were: (i) presence of any morbidity or disease that alters plasma levels of ROCK (e.g., arterial hypertension, dyslipidemia, insulin resistance, smoking or alcohol consumption habits, renal or liver dysfunction, neoplasia, and chronic respiratory and cardiac diseases) and (ii) use of anti-hypertensive, anorexic, anti-depressant, and/or antibiotics medication. In addition, a control group of healthy and non-active, sedentary subjects (*n* = 21) was included. The study was approved by the Ethics Committee of Pontificia Universidad Católica de Chile in observance of the Declaration of Helsinki on experimentation in human beings (project nº 16082603). Written informed consent was obtained from the subjects prior to any procedure.

**Figure 1:**
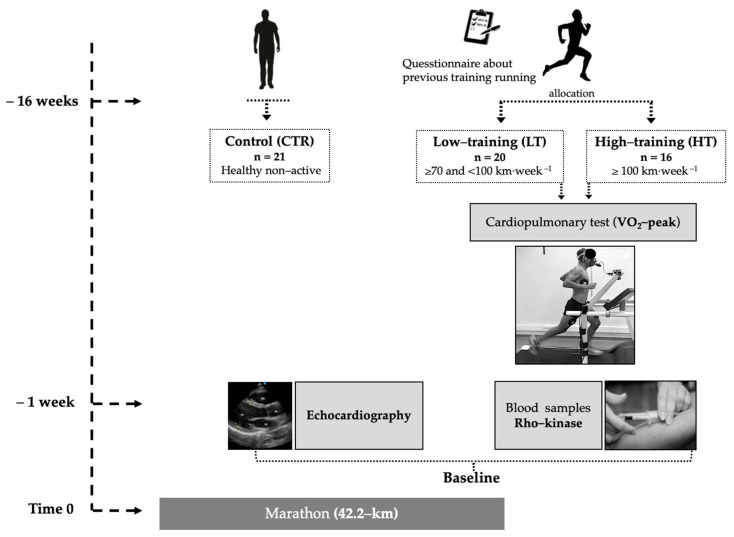
Study design scheme.

The study was cross-sectional with single blinding in researchers responsible for analyzing the serum samples and performing the echocardiographic reports and statistical analysis; they did not know to which group each subject belonged. After completing an ad hoc questionnaire with open questions concerning sports history, previous injuries, and time availability to training, LDRs were allocated to groups according to feasible weakly exercise volume during the training period before the marathon race. Running 100 km per week as a maximum exercise volume was adopted as a limit to separate the groups; this criterium has been used previously by our research group [10]. Thus, some LDRs were allocated to a high-training group (HT ≥ 100 km·week^−1^, *n* = 16) and a low-training group (LT ≥ 70 and <100 km·week^−1^, *n* = 20). Figure 1 shows the study design and assessments performed.

Through a venipuncture of the antecubital fossa, peripheral blood mononuclear cells were extracted to determine ROCK activity one week before the marathon competition. ROCK activity was determined in mononuclear cells, using protocols previously described [16]. Briefly, ROCK activation is determined by measuring the phosphorylation of a direct ROCK target, the myosin phosphatase target subunit 1 (MYPT1) of the myosin light chain phosphatase of the light chain of myosin, by Western blot. The antibodies used were anti-myosin phosphatase target subunit 1 (anti-MYPT-1 antibody, rabbit polyclonal, 1/500 cell signaling, Cat 2634) and anti-p-MYPT-1 antibody (phospho-MYPT1-Thr 853 rabbit polyclonal, 1/500, cyclex, Cat CY-P1025). ROCK activity was expressed by the quotient between phosphorylated (p-MYPT1) and total MYPT1 (t-MYPT1). We previously showed in an experimental model that ROCK activity in circulating leukocytes reflects activation of this signaling pathway in the myocardium and aortic wall [22]. Also, troponin-I levels were measured pre- and post-marathon race by a commercially available ELISA kit (CARD-I-KIT ELISA Troponin I, Labmaster, Finland) on an ELISA reader (Tecan-spectra, Austria), and calculated (ng·mL^−1^) as instructed by the manufacturer.

Transthoracic echocardiography (TTE) was performed in all participants using Vivid I echocardiography equipment (General Electric Healthcare, Horten, Norway) with a 2.5/5 MHz sector transducer. Traditional views were acquired from the windows: parasternal, apical, and subcostal for the quantification of the left and right heart chambers according to the American Society of Echocardiography [23]. LV systolic function was assessed by the ejection fraction (EF), calculated by the Simpson’s method. LV diastolic function was evaluated using transmitral filling waves and mitral annulus tissue Doppler. LV mass was calculated with the linear method (Devereux’s formula) as follows: 0.8 × 1.04 × [(IVS + LVID + PWT) ^3^ – LVID ^3^ ] + 0.6 g [23]. Also, LV longitudinal deformation (longitudinal strain) was carried out using four, three, and two chamber views optimized to achieve >60 frames per second. Images were stored for further analysis by an expert, blinded echocardiographer using the manufacturer’s software (EchoPAC, version BT12; GE Healthcare, Horten, Norway).

The physical performance of LDRs was assessed by the cardiopulmonary test (peak aerobic capacity, $\dot{V}$O_2_-peak) at the end of the “optimal phase” training period. All LDRs were instructed not to perform physical activity 48 h before the measurement and avoid intakes of alcohol, caffeine, or other stimulants and food for at least three hours before. The $\dot{V}$O_2_-peak test was measured on a treadmill ergometer (HP Cosmos, Traunstein, Germany) until voluntary exhaustion, despite oral breathing (respiratory quotient, 1.20 ± 0.05). The exercise protocol consisted of a 3 min rest, a 5 min warm-up (8 km·h^−1^), and a subsequent increase of 2 km·h^−1^ every 150 s, until all criteria for stopping the test were met. Ventilatory data were analysed breath-by-breath using open-circuit spirometry and were expressed under standard temperature, pressure, and dry (STPD) conditions (MasterScreen CPX, Jaeger^TM^, Würzburg, Germany). Before each test, the gas analyser and the volume transducer were calibrated according to the manufacturer’s instructions.

The normality of the data was evaluated using the Shapiro–Wilk test. The ANOVA and Student *t*-test were used to compare groups. The Pearson correlation test was used for assessing the association between ROCK activity levels and echocardiographic parameters linked to the cardiac remodelling process. The statistical software used was GraphPad Prism 8.0 (GraphPad Software Inc., San Diego, CA, USA). A value of *p* < 0.05 was considered statistically significant. To evaluate statistical power of the study a post-hoc power analysis was performed [24].

## 3. Results

Fifty-seven participants were consecutively included (age 37.4 ± 6.1 years). The LDRs achieved values of $\dot{V}$O_2_-peak according to their health status (LT: 52.5 ± 8.1 mL·kg^−1^·min^−1^ vs. HT: 58.5 ± 5.3 mL·kg^−1^·min^−1^; *p* = 0.02). The HT group completed the marathon race in the least time (231 ± 39 vs. 197 ± 33 min vs. 231 ± 39 LT, *p* = 0.03). Table 1 shows the participants’ characteristics.

**Figure 2:**
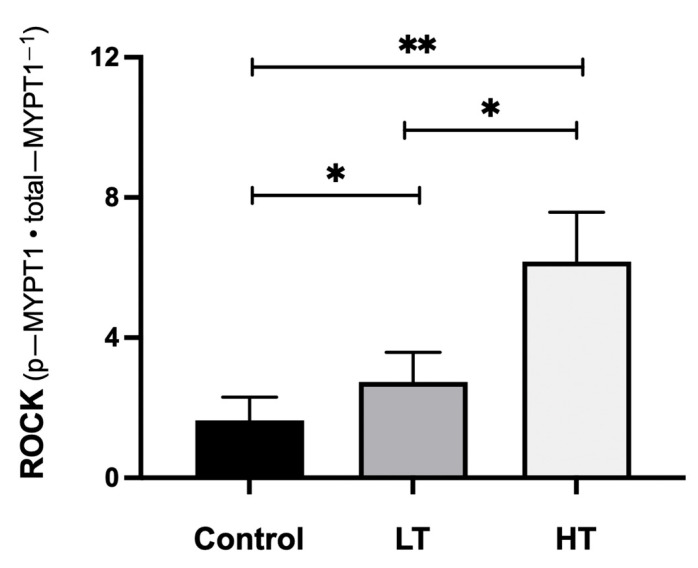
ROCK activity levels by group. Abbreviations: ROCK: Rho kinase activity expressed as p-MYPT1/t-MYPT1 ratio; LT: low training (≥70 and <100 km·week^−1^); HT: high training (≥100 km·week^−1^). * *p* < 0.01. ** *p* < 0.001.

The activity of ROCK was different between the groups. HT showed highest ROCK activation (6.17 ± 1.41 vs. 2.74 ± 0.84 LT (*p* = 0.002), and vs. 1.64 ± 0.66 CTR (*p* = 0.001); Figure 2). Post-hoc power analysis showed that differences between HT and LT, and HT and CTR groups had 100% power, whereas between LT and CTR group they had 99.6% power.

Regarding the quantification of the cardiac chambers by TTE, the results are shown in Table 2.

**Figure 3:**
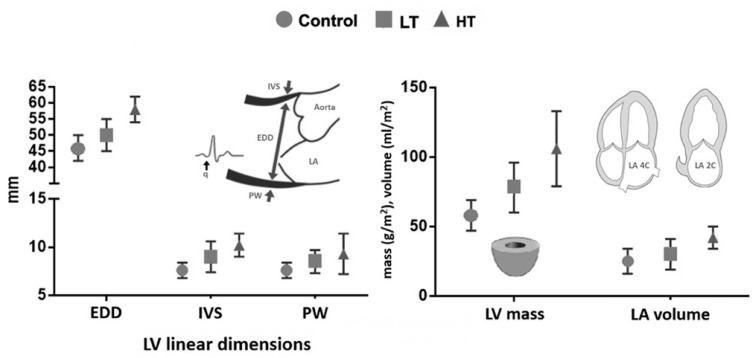
Quantification of left heart chambers. Abbreviations: LT: low training (≥70 and <100 km·week^−1^); HT: high training (≥100 km·week^−1^); EDD: end diastolic diameter; IVS: inter ventricular septum; PW: posterior wall; LV: left ventricle; LA: left atrium.

The HT group showed significantly larger LV linear dimensions than the other groups. Also, they showed a significantly increased LV mass index and LA volume index (Figure 3). LV diastolic function and right ventricle parameters were similar between groups.

**Figure 4:**
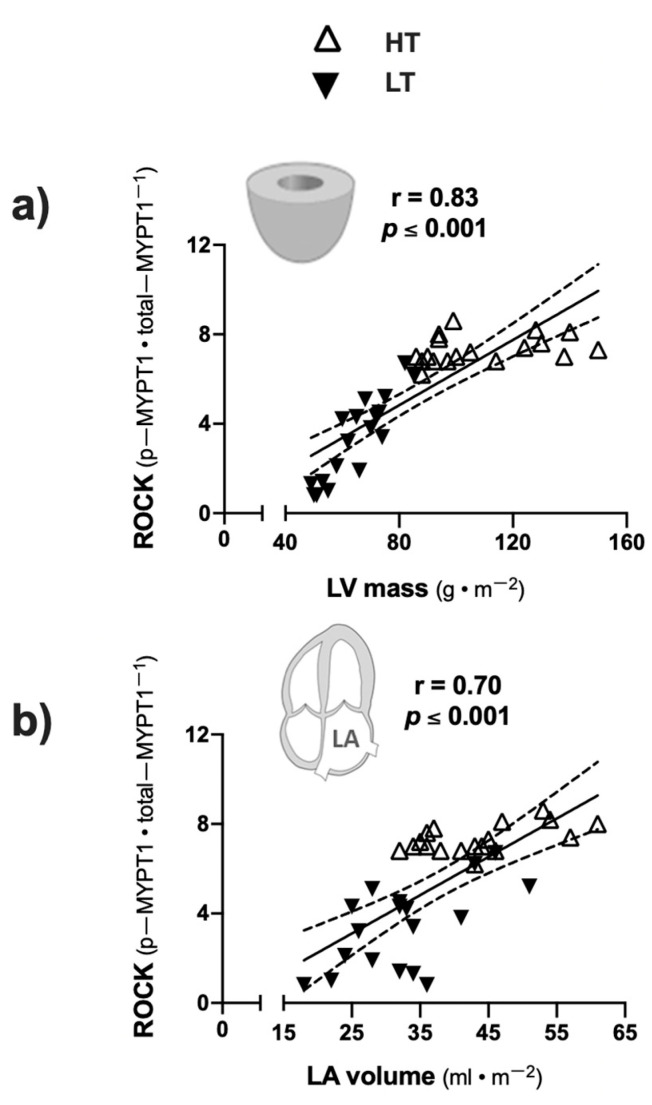
Correlation between ROCK activity and heart remodeling parameters. (**a**) ROCK activity (expressed as p−MYPT1/t−MYPT1 ratio) and LV mass index; (**b**) ROCK activity and LA volume index. Abbreviations: LV: left ventricle; LA: left atrium.

Among LDRs, a direct correlation between ROCK activation in circulating leukocytes, measured by the p-MYPT1/t-MYPT1 ratio, and left cardiac remodelling, evaluated by LV mass index (Figure 4a) and LA volume index, was found (Figure 4b).

## 4. Discussion

The main results of this study are that in LDRs, ROCK activation levels in circulation leukocytes were correlated with LV and LA remodelling, as evaluated by indexed LV mass and LA volume. It is expected that with a greater volume of LV cavity the deformation will be less, but we did not find that difference between our groups, probably due to the small differences in the magnitude of left ventricular size between the groups. Also, HT runners showed higher ROCK activity in circulating leukocytes than LT runners and physically healthy, non-active participants. Also, the HT group showed more significant LV diastolic diameter, LV wall thickness, and LV indexed mass, with normal deformation properties and systo-diastolic function; also, in the HT group LA volume was bigger, but with normal filling pressures.

Previous reports revealed that athletes who participate in competitive endurance sports show a heart remodelling process similar to our findings. Athletes showed an increased LA volume and LV mass with normal systolic and diastolic function; these structural changes were more pronounced in those with a more intensive training protocol [6,12,13]. A meta-analysis in athletes with high training regimes showed that acute and prolonged exercise was associated with larger LV volumes and a relative lower LV ejection fraction post-intensive exercise [25]. In this study, the HT group showed a greater left cardiac remodelling process, which potentially gives athletes better performance in highly demanding races [10]; however, these changes could be related to a theoretically excessive and deleterious remodelling process [12], which is in keeping with other reports showing inflammatory and cell remodelling biomarker elevation in athletes, whose complete significance is not fully clarified [22,23].

The ROCK signaling pathway participates in cell survival and cardiac hypertrophy, cardiac fibrosis, and cell apoptosis [15]. Some athletes may develop an extreme cardiac remodelling process, including atrial structure and function changes [12,13,26], worse ventricular performance post-effort [6], and appearance of arrhythmias in long-term follow-up [27,28]. Regarding the physiopathology of previous processes, the ROCK signaling pathway could play an important role in electrical property changes in atrial tissue (connexin expression), as Chen et al. (2018) showed while studying the LA appendage in patients with and without atrial fibrillation [29]. In our study, the HT group showed higher left cardiac cavity remodelling, ROCK activity, and sports performance than other groups; these pathways are probably related to cell survival in stress, and cardiac remodelling allows athletes to better adapt and performance extreme efforts, but there could be an individual point for each subject in which a poor adaptation may be observed. This “point” could lead to an extreme cardiac remodelling process and the risk of future arrhythmias [6] and cardiac dysfunction.

Rho kinase activity is associated with several mechanisms of cardiac injury including ischemic/reperfusion phenomenon [30]. A recent meta-analysis of animal models of myocardial ischemia/reperfusion injury showed that fasudil, a Rho kinase inhibitor, exerts a cardioprotective effect including lowering of troponin-I elevation [31]. Multiple protocols have assessed troponin levels post-different intensities of exercise, but the results have different results and there have been different interpretations on the mechanism of troponin release, which probably depends on multiple individual mechanisms of adaptation to exercise [26,27,32]. In our protocol we did not find troponin-I elevation post-marathon race (troponin-I < 0.156 ng·mL^−1^ in all subjects).

Athletes showed a systemic and cellular adaptation to mechanical, inflammatory, and metabolic stress caused by regular and intense exercise training that generated adaptation mechanisms, such as cardiac remodelling and changes in cardiac biomarker expression [33], so that more trained athletes could perform higher workloads, resulting in better performance in highly demanding competitions; however, some subjects could experience extreme and potentially non-adaptive changes [6,13]. Maladaptive changes could be partially explained by experimental models of prolonged intense exercise that showed an increased expression of tumour growth factor-β1 (TGF-β1) in atrial and right ventricular tissue related to myocardial stiffness and myocardial fibrosis [34]. Genetic influences in ROCK activation cannot be ruled out in the formation of cardiac fibrosis associated to a remodelling process induced by intense exercise and regular physical training load. In this regard, normal rodents with genetically higher angiotensin-converting enzyme levels have increased ROCK cascade activation simultaneously in the heart and circulating leukocytes, and they develop higher LV fibrosis levels in response to isoproterenol [35].

Therefore, we suggest that these processes of conditioning and adaptation to continuous effort could result in a “maladaptation” in individual athletes over time, triggering an adverse remodelling process which would be explained by the concept of “hormesis”, that is defined as an adaptive cellular response to stressors resulting in a biphasic dose-response—low doses are related to a beneficial adaptation while high doses result in an adverse effect in some specific subjects. Currently, it is unknown precisely what the real meaning of these changes and the behavior of cardiac remodelling biomarkers in apparently non-pathological conditions is and whether they predispose to and partly explain the development of arrhythmias in this subgroup. Moreover, ROCK activity in circulating leukocytes, which reflects activation of this pathway in the myocardium [22], is related to adverse cardiac remodelling in patients with hypertension and cardiac failure [17,18] making the interpretation of our findings more complex.

An aspect that could interfere with our results is the seasonal variations in corporal composition and hydration status during the training period in competitive athletes, affecting plasmatic volume (and cardiac output) and hemodynamic variables that could interfere in the echocardiography measurements obtained in LDRs [36].

One of the main limitations of our study was the reduced number of participants recruited. Therefore, some of the results should be taken cautiously as due to the relatively small sample size, the data may not have the statistical power to expose small effects of training on ROCK activity and cardiac remodelling, possibly resulting in a type II error. However, post-hoc analysis of ROCK activity data showed that the differences had almost 100% power, suggesting that the sample size of this work is sufficient to assess differences in this parameter. Other limitations were the exclusive participation of male athletes, that our conclusions are potentially applicable exclusively to long-distance runners, and that our results are applicable only to the Caucasian ethnic group. Also, the presence of myocardial fibrosis with cardiac resonance was not evaluated. We assumed that athletes had normal systo-diastolic function and normal deformation (global longitudinal strain) of the left ventricle, only with echocardiographic assessment. The applicability of a questionnaire with open questions to assign athletes to runner groups (HT or LT) also is a limitation, mainly because we did not objectively measure the different levels of physical activity completed by runners during the weeks before allocation, for example, reviewing sports activities completed and loaded into training software (e.g., TrainingPeaks™, EnduranceTool™, etc.).

There is no doubt that moderate exercise training leads to good cardiovascular health and longevity [1]. However, there is increasing evidence that more intense exercise can produce potentially excessive cardiac remodelling and arrhythmic events in the long-term follow-up. So, it is essential to have available and accessible clinical tools and biomarkers that help identify those individuals at a greater risk.

## 5. Conclusions

In male long-distance competitive runners, the load of exercise implicated in marathon training (overload cardiac volume) is associated with ROCK activity levels and a left cardiac remodelling process assessed by echocardiography.

## Figures and Tables

**Table 1:** Participant’s characteristics.

	Groups			
Variables	Control (*n* = 21)	LT (*n* = 20)	HT (*n* = 16)	*p*-Value
Age (years)	35 ± 4	39 ± 5	37 ± 6	0.32
Height (cm)	175 ± 6	174 ± 6	172 ± 7	0.47
Weight (kg)	72 ± 9	73 ± 8	69 ± 8	0.09
Body surface (m^2^)	1.89 ± 0.13	1.88 ± 0.12	1.82 ± 0.13	0.08
Creatinine (mg·dL^−1^)	0.99 ± 0.11	0.97 ± 0.10	0.98 ± 0.09	0.63
Hematocrit (%)	42 ± 3	43 ± 3	43 ± 2	0.87
Sodium (mEq/L)	142 ± 2	142 ± 2	142 ± 3	0.44
AST (U/L)	26 ± 7	28 ± 8	29 ± 9	0.67
Uric acid (mg/dL)	5.2 ± 0.8	5.0 ± 0.9	5.6 ± 0.9	0.17
$\dot{V}$O_2_-peak (mL·kg^−1^·min^−1^)	-	52.5 ± 8.1	58.5 ± 5.3	0.02 *
Running experience (years)	-	15 ± 3	17 ± 3	0.81
Time training per week (h)	-	14 ± 2	19 ± 2	0.01 *
Training intensity (%HR máx., 220-age)	-	82 ± 2	81 ± 3	0.78

Data are reported as the mean ± SD; Student’s *t* test, * *p* < 0.05. Abbreviations. CTR: Control; LT: Low training (≥70 and <100 km·week^−1^); HT: High training (≥100 km·week^−1^); AST: Aspartate amino transferase; $\dot{V}$O_2_-peak: Peak oxygen consumption.

**Table 2:** Heart chambers quantification.

	Groups			
	Control (*n* = 21)	LT (*n* = 20)	HT (*n* = 16)	*p*-Value
**Left Cardiac Cavities**				
Interventricular septum (mm)	7.6 ± 0.8; (10.5)	9.0 ± 1.6; (17.7)	10.2 ± 1.2 *; (11.89	<0.001
Posterior wall (mm)	7.6 ± 0.8; (10.5)	8.5 ± 1.2; (14.1)	9.3 ± 2.1 *; (22.6)	0.01
LVEDD (mm)	46 ± 4; (8)	50 ± 5; (10)	58 ± 4 *; (6)	0.04
LVESD (mm)	30 ± 3; (10)	30 ± 4; (13)	33 ± 5; (15)	0.40
Ejection fraction (%)	57 ± 4; (7)	55 ± 6; (10)	54 ± 3; (5)	0.11
LV mas index (g·m^−2^)	58 ± 11; (18)	78 ± 18; (23)	106 ± 27 *; (25)	<0.001
LA diameter (mm)	33 ± 4; (12)	34 ± 3; (8)	36 ± 4; (11)	0.22
LA area (cm^2^)	19 ± 5; (26)	22 ± 4; (18)	25 ± 3 *; (12)	<0.001
LA volumen index (mL·m^−2^)	25 ± 9; (36)	30 ± 11; (36)	42 ± 8 *; (19)	<0.001
Global LV longitudinal strain (%)	−21.0 ± −2.0; (9.5)	−19.6 ± −1.6; (8.2)	−19.5 ± −2.4; (12.3)	0.11
E wave (cm·s^−1^)	77 ± 15; (19)	84 ± 12; (14)	78 ± 13; (17)	0.21
A wave (cm·s^−1^)	48 ± 16; (33)	53 ± 10; (19)	50 ± 12; (24)	0.43
Deceleration time (ms)	200 ± 66; (33)	229 ± 65; (28)	233 ± 65; (28)	0.18
e’ lateral (cm·s^−1^)	15.0 ± 1.8; (12.0)	15.0 ± 2.5; (16.7)	15.0 ± 2.3; (15.3)	0.70
e’ medial (cm·s^−1^)	11.0 ± 1.8; (16.4)	10.0 ± 2.0; (20.0)	10.0 ± 2.0; (20.0)	0.75
**Right Ventricle**				
TAPSE (mm)	25.4 ± 3.3; (12.9)	25.6 ± 4.7; (18.3)	25.8 ± 3.0; (11.6)	0.16
FAC (%)	52.5 ± 3.9; (7.4)	57.3 ± 4.6; (8.0)	56.4 ± 3.7; (6.6)	0.07

Data are reported as the mean ± SD; coefficient of variation (CV). ANOVA test (HT vs. other groups), * *p* < 0.05. Abbreviations: CTR: Control; LT: Low training (≥70 and <100 km·week^−1^); HT: High training (≥100 km·week^−1^); LVEDD: left ventricular-end diastolic diameter; LVESD: left ventricular-end systolic diameter; LV: left ventricle; LA: left atrium; é: mitral annulus tissue Doppler; TAPSE: tricuspid annulus plane systolic excursion; FAC: fractional area change.
